# Effect of BuShen JiangZhi Recipe on Atherosclerosis in ApoE^−/-^ Mice by Regulating the Expression of Anpep via mmu_circRNA_22187

**DOI:** 10.1155/2021/4738264

**Published:** 2021-09-03

**Authors:** Li Yan, Qingling Jia, Hui Cao, Pingyi He, Sanli Xing, Yan Huang, Chuan Chen, Dingzhu Shen

**Affiliations:** Shanghai Geriatric Institute of Chinese Medicine, Shanghai University of Traditional Chinese Medicine, Shanghai 200031, China

## Abstract

The BuShen JiangZhi (BSJZ) recipe is a Chinese medicine compound with the effect of tonifying the kidney, replenishing essence, and lowering blood fat to unblock vessels. The purpose of this study is to explore whether the mechanism of BSJZ for effective intervention in the treatment of AS is related to mmu_circRNA_22187 and aminopeptidase N (Anpep). ApoE^−/−^ mice were induced by a high-fat diet to replicate the AS model. 24 ApoE^−/−^ mice were randomly divided into model group (group M), BSJZ group (group BS), and 12 C57BL/6 mice of the same genetic background and same weeks of age as the normal control group (group C). Mice in the BS group were given an aqueous solution of BSJZ by gavage, while mice in groups C and *M* were given the same volume of distilled water. HE and Oil Red O staining were used to detect the pathomorphology and lipid accumulation of mouse aortic sinus. Arraystar version 2.0 mouse circRNA chip was used to scan with Agilent Scanner G2505C, and the differential circRNAs expression profile of mice aorta was obtained. Scatter plot, volcano plot, and cluster map, respectively, visualized the differentially expressed circRNAs, as well as the types of circRNAs and the chromosomes' distribution, screened and compared the differentially expressed circRNAs intersection between groups by Venny software, and then combined ceRNA bioinformatics analysis to construct a ceRNA network. The results showed that BSJZ could significantly reduce the area of AS plaque and lipid accumulation in the aortic sinus of ApoE^−/−^ mice induced by a high-fat diet. The bioinformatics analysis showed that mmu_circRNA_22187 may be a key circRNA of BSJZ intervention in the treatment of AS. Compared with group C, the expressions of Anpep mRNA and protein were upregulated in group M. After the intervention of BSJZ, the expressions of Anpep mRNA and protein were downregulated. Therefore, BSJZ could effectively treat AS which might be related to the regulation of mmu_circRNA_22187 and Anpep.

## 1. Introduction

Atherosclerosis (AS) is a complex multifactorial disease, characterized by lipid deposition in the arterial wall, which is the key pathological basis for the occurrence of cardiocerebrovascular diseases [1]. It is reported that about 50% of the risk of AS is closely related to heredity [2]. Circular RNAs (circRNAs), a new class of endogenous noncoding RNA, is characterized by the closed-loop structure formed by the covalent connection between its 3′ end and 5′ end, which has stronger endonuclease tolerance and higher stability than linear mRNA [3]. CircRNAs have a variety of biological functional mechanisms in human diseases, including acting as a miRNA sponge, interacting with proteins, regulating gene splicing or transcription, translating proteins or peptides, and epigenetic regulation [4]. In recent years, circRNAs play an important role in the pathogenesis of cardiovascular disease and are closely related to the development of AS. Related studies have shown that circRNAs have been identified in human coronary artery, which can be used as a diagnostic marker or therapeutic target for coronary artery disease [5]. It is shown that the circRNA hsa_circ_0003575 is significantly upregulated in human umbilical vein endothelial cells (human umbilical vein endothelial cells, HUVECs) induced by oxidized low-density lipoprotein (ox-LDL) and silencing hsa_circ_0003575 can promote the proliferation and angiogenesis of HUVECs [6]. In vivo, it has been reported that the expression of CircHIPK3 is downregulated in the aorta of ApoE^−/−^ mice fed with a high-fat diet, and HUVECs autophagy is induced by targeting miR-190b/ATG7, which plays an important role in the pathogenesis of AS [7]. Therefore, an in-depth study of AS from the genome level is of great significance for the diagnosis and treatment of the disease.

In recent years, Traditional Chinese Medicine has been proved some good curative effects in multiway, multitarget, and multilink intervention in the treatment of AS [8]. BSJZ is composed of Tusizi, Gouqizi, Shayuanzi, Nüzhenzi, Fupenzi, and Heidou, 10 g each, which has the function of tonifying the kidney, replenishing essence, and lowering blood fat to unblock vessel. Our previous studies showed that BSJZ can effectively improve lipid metabolism disorders, promote autophagy, and regulate the levels of inflammatory factors, thereby effectively intervening in the treatment of AS [9, 10]. However, it is not clear whether the key mechanism of BSJZ anti-AS involves related circRNA changes and how it plays a role in the pathological process of AS.

In the current study, we replicated the AS model in ApoE^−/−^ mice induced by a high-fat diet. CircRNA microarray was used to identify differentially expressed circRNAs, the differentially expressed circRNAs were analyzed by bioinformatics, the differentially expressed circRNAs between groups were used for Venn intersection, and a ceRNA network was established to obtain mRNAs related to circRNAs. The differential expression of circRNAs (mmu_circRNA_20264, mmu_circRNA_22187, mmu_circRNA_32161, mmu_circRNA_43213, and mmu_circRNA_43970) and Anpep mRNA was detected by quantitative reverse transcriptase-polymerase chain reaction (qRT-PCR), and the expression of Anpep protein was analyzed by Western blot. All of these were hoping to provide new evidence for the mechanism of BSJZ in the intervention and treatment of AS.

## 2. Materials and Methods

### 2.1. Experimental Animals

Specific Pathogen Free (SPF) grade 12-week-old male ApoE^−/−^ mice (SCXK (Jing) 2016-0006) and male C57BL/6 mice of the same genetic background and the same age (SCXK (Zhe) 2018-0001) were purchased from Beijing Vital River Lab Animal Technology Co., Ltd. They were fed in a single cage in the SPF animal laboratory of the Experimental Animal Center of Shanghai University of Traditional Chinese Medicine, with an ambient temperature of (24 ± 1)°C, relative humidity of 50%–70%, and a light-dark cycle of 12/12 h. It has passed the experimental animal welfare and ethics review of Shanghai University of Traditional Chinese Medicine (ethics number: PZSHUTCM181130001).

### 2.2. Modeling and Grouping

Twenty-four 12-week-old male ApoE^−/−^ mice were fed with a normal diet for 1 week and randomly divided into 2 groups: ApoE^−/−^ mice + high-fat diet group (group *M*, *n* = 12) and ApoE^−/−^ mice + high-fat diet + BSJZ group (group BS, *n* = 12). The high-fat diet formula was composed of mouse normal diet, 21% fat, and 0.5% cholesterol; the same genetic background and the same age C57BL/6 mice were used as the control group (group C, *n* = 12), and they were fed with a normal diet for mice. Mice in the BS group were given the aqueous solution of BSJZ, and BSJZ granules were directly dissolved in distilled water. Calculated with reference to the 60 kg adult body weight and mouse equivalent dose formula, the gavage dose and frequency of BSJZ aqueous solution were 1644 mg/kg and 0.2 ml/mouse, once a day; mice in groups C and *M* were given an equal volume of distilled water once a day, and the intervention period of each group was 12 weeks.

### 2.3. Drugs and Reagents

BSJZ is composed of 10 g each of Tusizi (Semen Cuscutae), Gouqizi (Fructus Lycii), Shayuanzi (Semen Astragali Complanati), Nüzhenzi (Fructus Ligustri Lucidi), Fupenzi (Fructus Rubi Chingii), and Heidou (Semen Sojae Atricolor), and the corresponding doses of raw herbal granules are 0.5 g, 4 g, 1 g, 1 g, 1 g, and 0.5 g (Jiangyin Tianjiang Pharmaceutical Industry Co., Ltd. Jiangsu, China, 1702005, 1601007, 1703040, 1704007, 1702030, and 1702036). 12% SDS-PAGE lower gel premix (Beyotime, P0672), SDS-PAGE upper gel premix (Beyotime, P0683), BCA protein concentration determination kit (Beyotime, P0010), SDS-PAGE protein loading buffer solution (5×) (Beyotime, P0015 L), High-sig ECL Western Blotting Substrate (Shanghai Tianneng Technology Co., Ltd., 180-5001), prestained protein maker (Thermo Fisher Scientific, 26616), rabbit anti-CD13/APN (Signaling Technology, 32720S), rabbit anti-GAPDH (Signaling Technology, 5174S), HRP-labeled goat anti-rabbit secondary antibody (Signaling Technology, 7074P2), DEPC water (Beyotime, R0021), and SYBR® Green Real-Time PCR Master Mix (TOYOBO Company, 952200) were used.

### 2.4. Hematoxylin-Eosin (HE) and Oil Red O Staining

Mice were anesthetized with 2% pentobarbital sodium intraperitoneally and perfused with 4% paraformaldehyde. The heart was quickly taken with the aortic tissue and preserved in 10% formalin for paraffin and frozen sections for HE staining (paraffin sections) and Oil Red O staining (frozen sections) to observe the pathological morphology and lipid accumulation in the aortic sinus of mice, respectively. The HE stained images were observed and acquired using an optical microscope. The Oil Red O stained images were observed and acquired by a scanner [10]. The images were analyzed semiquantitatively using Image-Pro Plus: plaque area ratio = plaque area/total aortic sinus lumen area; red-stained lipid area ratio = red-stained lipid area/aortic sinus lumen area.

### 2.5. CircRNA Microarray Analysis

Total RNA was extracted from mouse aortic tissue using TRIzol LS Reagent (Invitrogen life technologies), RNA concentration and purity were determined by NanoDrop® ND-1000, and the RNase R-treated samples were subjected to labeling reactions and array hybridization [11]. The data were scanned with an Agilent Scanner G2505 C (Agilent, Santa Clara, CA, USA) using an Arraystar version 2.0 mouse circRNA microarray, the raw data were read by Agilent Feature Extraction software (v11.0.1.1) (Agilent, Santa Clara, CA, USA.), and the *R* software limma package normalizes the qualified data of quality control and analyzes the standardized gene chip data to obtain differential circRNAs expression profile. CircRNAs were identified to be differentially expressed (fold change >1.5; *P* < 0.05) [12].

### 2.6. Bioinformatics Analysis

Because of the variability between samples, we first normalized the raw expression values of circRNA microarray data from each group of mouse aorta and used box plots to show the intensity distribution pattern of the standardized data, analyzed the data to obtain the expression profiles of differentially expressed circRNAs in mouse aorta, and visualized them. Venny analysis (https://bioinfogp.cnb.csic.es/tools/venny/index.html) was performed to screen the intersection of differentially expressed circRNAs between groups and then ceRNA bioinformatics analysis was performed to determine the distribution of circRNAs on chromosomes. The ceRNA network was constructed using Cytoscape 3.6.1 [13].

### 2.7. Quantitative Real-Time Polymerase Chain Reaction Analysis

Total RNA (2 *μ*g) was treated with RNase R (Epicentre) for 15 min at 37°C to remove linear transcripts. Treated RNA and total RNA (2 *μ*g) were reverse transcribed to homologous cDNA using PrimeScript RT Master Mix (Perfect Real Time, TaKaRa), and total RNA (2 *μ*g) was synthesized with miRcute miRNA First-Strand cDNA Synthesis Kit (TIANGEN) to reverse transcribe the total RNA (2 *μ*g). The cDNA was then analyzed by qRT-PCR using EvaGreen qPCR Mastermix-s (abm) and Light Cycler 480 II system (Roche). GAPDH was used as an internal control for circRNA and mRNA (see Table 1 for details of primer sequences) and the relative expression levels of RNA were calculated using the 2^−ΔΔ^CT method [11].

### 2.8. Western Blot Analysis

Mice were anesthetized with 2% pentobarbital sodium intraperitoneally, the thoracic to abdominal aortic segments were taken, the total protein of mouse aorta was extracted and quantified by BCA assay, and the protein was separated by SDS-PAGE electrophoresis (80 V, 30 min; 120 V, 90 min), transferred to PVDF membrane by wet transfer (270 mA, 90 min), and incubated at room temperature for 2 h in 5% Skim milk which was blocked at room temperature for 2 h [14]. After closure, primary antibodies Anpep (1 : 1000) and GAPDH (1 : 2000) were added and incubated overnight at 4°C. Wash the membrane for 10 min × 3 times, add HRP-labeled secondary antibody (1 : 2000), incubate for 1 h at room temperature, and wash the membrane for 10 min × 3 times with TBST. The membrane was incubated for 1 h at room temperature and washed 10 min × 3 times with TBST. The luminescent solution A was mixed 1 : 1 with the luminescent solution B according to the Tanon^TM^ High-sig ECL Western Blotting Substrate kit instructions and then quickly developed on the PVDF membrane. The iBright FL1000 Imaging System (Thermo Fisher Scientific, Inc.) protein blotting intelligent imaging system was used for scanning. The integrated absorbance (IA = average optical density *x* area) of the protein bands was analyzed using Image *J* software, and the IA/GAPDH IA values of the target proteins were used to reflect the relative expression levels of the proteins.

### 2.9. Statistical Analysis

SPSS 24.0 was used for statistical analysis, experimental data were expressed as mean ± standard deviation ($\bar{x}$ ± *s*), and data were analyzed using one-way ANOVA followed by least significant difference (LSD) for comparison among groups. Graph Pad Prism, Adobe Illustrator, Adobe Photoshop, and Cytoscape were used for drawing. The test level was taken as two-sided *α* = 0.05, and *P* < 0.05 was considered a statistically significant difference.

## 3. Results

### 3.1. Pathological Morphology of Aortic Sinus

**Figure 1:**
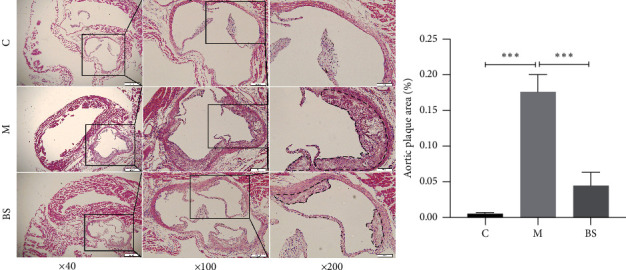
Pathological morphology of aortic sinus (HE staining: ×40, ×100, and ×200); ^∗∗∗^*P* < 0.001, versus group C; ^∗∗∗^*P* < 0.001, versus group M. C: control group; M: model group; BS: BSJZ group.

No significant AS plaque was seen in the aortic sinus of mice in group C; compared to group C, the area of AS plaque in the aortic sinus of mice in group *M* was significantly increased and cholesterol crystals were visible (*P* < 0.001) (Figure 1); compared with group *M*, the area of AS plaque in the aortic sinus of mice in group BS was significantly reduced (*P* < 0.001) (Figure 1).

### 3.2. Lipid Accumulation of Aortic Sinus

**Figure 2:**
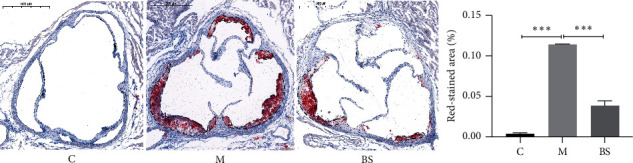
Lipid accumulation of aortic sinus (Oil Red O staining: ×40); ^∗∗∗^*P* < 0.001, versus group C; ^∗∗∗^*P* < 0.001, versus group M. C: control group; M: model group; BS: BSJZ group.

There was no obvious red-stained lipid in the aortic sinus of mice in group C; lipid accumulation in the aortic sinus of mice in group *M* was significantly increased compared with group C (*P* < 0.001) (Figure 2); lipid accumulation in the aortic sinus of mice in group BS was significantly reduced compared with group *M* (*P* < 0.001) (Figure 2).

### 3.3. Identification of Aortic CircRNAs and Analysis of Their Differential Expression

**Figure 3:**
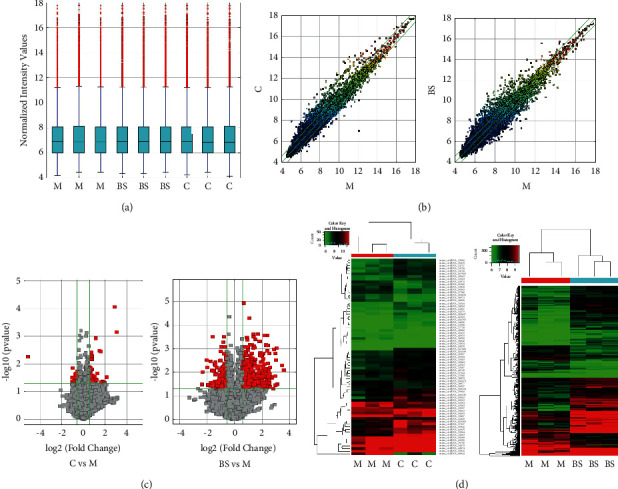
Identification of circRNAs in mouse aorta and their differential expression analysis: (a) intensity distribution after normalization of data; (b) scatter plot: the green line represents the multiple of difference, and the dots above and below the green line represent the circRNAs that are upregulated by 1.5 times and downregulated by more than 1.5 times, respectively; (c) volcano plot: two green vertical lines represent 1.5 times upregulation and 1.5 times downregulation, green horizontal lines indicate *P* value 0.05, and red squares represent statistically significant differentially expressed genes; (d) clustering plot: green and red represent low and high expression levels, respectively.

**Figure 4:**
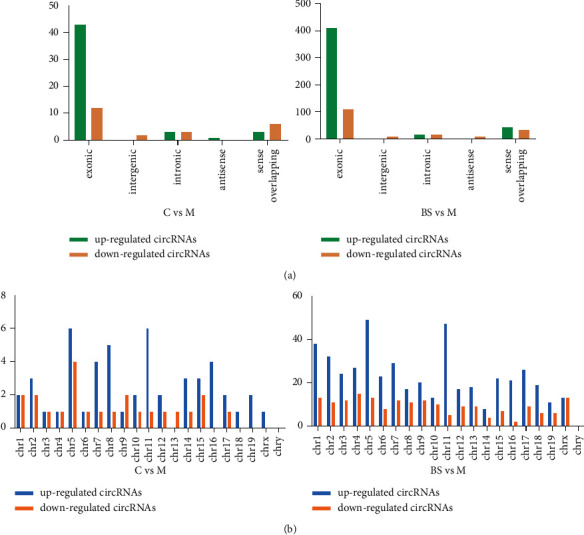
circRNA species and their chromosome distribution: (a) circRNAs species; (b) circRNAs chromosome distribution.

As shown in the figure below, the results indicate that the intensity distribution of circRNA microarray data was very close across groups (Figure 3(a)). The scatter plot (Figure 3(b)), volcano plot (Figure 3(c)), and cluster analysis plot (Figure 3(d)) further showed that the differential expression of circRNAs in mouse aorta was significant (*P* < 0.05, FC > 1.5). A total of 74 circRNAs were differentially expressed in *C* and group *M* (*C* vs *M*), 653 circRNAs were differentially expressed in group BS and group M (BS vs M), mostly distributed on chromosome 5 (Figure 4).

### 3.4. mmu_circRNA_22187 May Be a Key Target of BSJZ Intervention in the Treatment of AS

**Figure 5:**
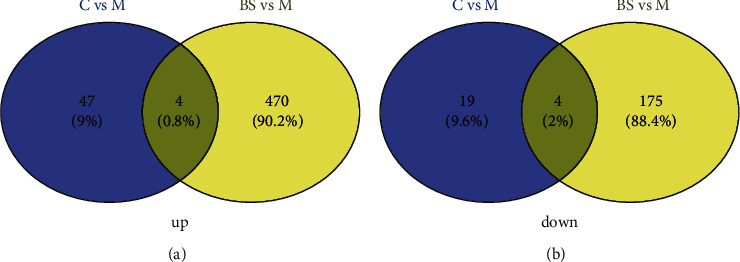
Venn diagram of differential expression of circRNAs in the aorta of mice in each group.

The differentially expressed circRNAs in mouse aorta were analyzed with Venny software to screen the differentially expressed circRNAs in each group for venn intersections. Each circle indicates the differential genes in the comparison groups (C vs *M*, BS vs M), the numbers in the overlapping areas indicate the number of shared differential genes between the corresponding multiple comparison groups, and the nonoverlapping regions indicate the differential genes specific to each comparison group. The intersection results showed that there were a total of 8 circRNAs (mmu_circRNA_43970, mmu_circRNA_001170, mmu_circRNA_38889, mmu_circRNA_33227, mmu_circRNA_22187, mmu_circRNA_ 43213, mmu_circRNA_20264, and mmu_circRNA_32161) upregulated or downregulated in comparison between groups (C vs M, BS vs M) (Figure 5).

**Figure 6:**
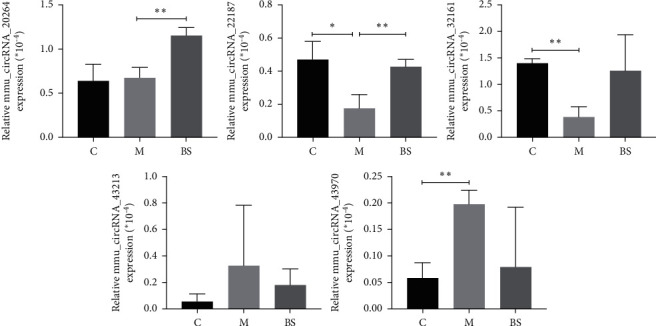
Qrt-PCR validation of differentially expressed circRNAs; ^∗∗∗^*P* < 0.01 and ^∗^*P* < 0.05, versus group C; ^∗∗^*P* < 0.01, versus group M. C: control group; *M:* model group; BS: BSJZ group.

Next, five differentially expressed circRNAs were obtained by ceRNA bioinformatics analysis, mmu_circRNA_20264, mmu_circRNA_22187, mmu_circRNA_32161, mmu_circRNA_43213, and mmu_circRNA_43970, and the construction of ceRNA network. The validation of qrt-PCR showed that the expression of mmu_circRNA_22187 and mmu_circRNA_32161 were downregulated (*P* < 0.05; *P* < 0.01), mmu_circRNA_43970 was upregulated (*P* < 0.01) in the aorta of high-fat diet-induced ApoE^−/−^ mice, while the expression level of mmu_circRNA_22187 was upregulated after the intervention of BSJZ (Figure 6), suggesting that mmu_circRNA_22187 may be a key target for effective intervention of BSJZ in the treatment of AS.

### 3.5. BSJZ May Regulate Anpep in the Treatment of AS via mmu_circRNA_22187

**Figure 7:**
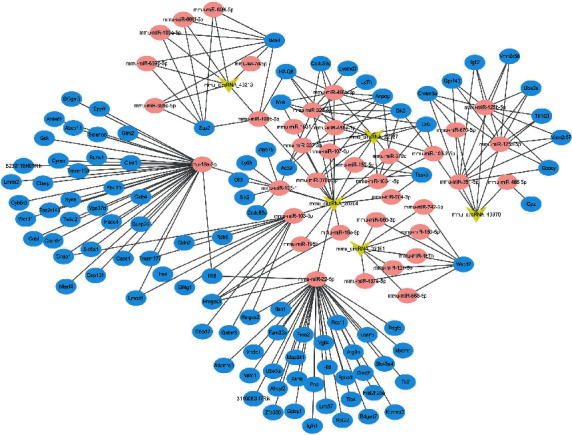
CircRNA-miRNA-mRNA network. The network consists of 5 circRNAs, 37 miRNAs, and 101 mRNAs (yellow markers represent circRNAs, pink markers represent miRNAs, and blue markers represent mRNAs).

**Figure 8:**
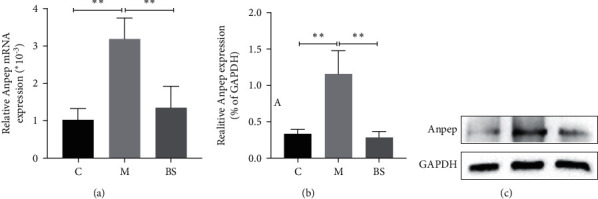
Anpep protein expression in the aorta of various groups of mice; ^∗∗∗^*P* < 0.01, versus group C; ^∗∗∗^*P* < 0.01, versus group M. C: control group; M: model group; BS: BSJZ group.

Based on the ceRNA network (Figure 7), we found that Anpep mRNA may be correlated with mmu_circRNA_22187. qRT-PCR results showed that, compared with group C, Anpep mRNA and protein expression were elevated in group *M* (*P* < 0.01), and after the intervention of BSJZ, Anpep mRNA (Figure 8(a)) and protein expression decreased (*P* < 0.01) (Figures 8(b) and 8(c)), suggesting that BSJZ may affect AS by regulating the expression of Anpep through mmu_circRNA_22187.

## 4. Discussion

CircRNA is a covalently closed circular RNA molecule with properties such as high stability, conservation, localization, and expression specificity [15]. With the wide application of RNA sequencing (RNA-seq) technology and bioinformatics predictions, a large number of circRNAs have been identified, among which microarrays are considered to be one of the effective tools for circRNA analysis [16]. In a clinical evaluation of circulating circRNA diagnostic biomarkers for stable coronary heart disease, it was found that plasma hsa_circ_0001445 in patients with coronary heart disease was strongly negatively correlated with the severity of AS [17]. In addition, hsa_cic_0001879 and hsa_cic_0004104 are significantly upregulated in the plasma of patients with coronary heart disease, which provides new clues for the potential of circRNA as a biomarker for the diagnosis of coronary heart disease [18]. At present, circRNA has the functions of acting as a microRNA (miRNA) sponge, regulating protein binding, regulating gene transcription and coding genes, and playing an important role in cardiovascular diseases [19]. AS, as the key pathological basis of cardiovascular and cerebrovascular events, is a complex disease involving multiple factors. According to reports, in THP-1 macrophage-derived foam cells induced by ox-LDL, hsa_circ_0028198/hsa_circ_0092317/XIST-miR-543 may inhibit ASPH mRNA expression through a competitive endogenous RNA (ceRNA) mechanism, thereby reducing AS lesions [20]. A study has shown that circRNA-0044073 promotes the proliferation and invasion of human vascular smooth muscle cells (HUVSMC) and human vascular endothelial cells (HUVEC) by targeting miR-107 and activating the JAK/STAT signaling pathway, thereby providing a potential therapeutic target for the treatment of AS [21]. Another study has also shown that the expression of circ-PTPRA in the serum of AS patients is upregulated by inhibiting miR-636 and upregulating the SP1 signal axis, which promotes cell proliferation and inhibits cell apoptosis, revealing that circ-PTPRA may be a marker and treatment target for AS patients [22]. Therefore, circRNA may play an important role in the diagnosis and treatment of AS.

Although the term “AS” is not directly mentioned in Chinese medicine, according to its clinical features, it can be classified into categories such as “rheumatism with blood vessel involved,” “chest discomfort,” “vertigo,” and “apoplexy.” At present, Chinese medicine has a significant effect in treating AS. According to the reports, Yiqi Huoxue Recipe can effectively regulate lipid metabolism in ApoE^−/−^ mice fed with a high-fat diet, inhibit IL-6/STAT3, improve inflammation, reduce liver toxicity, and thereby inhibit the progression of AS [23]. In a rat model of AS induced by vitamin D3 and ovalbumin and fed with a high-fat diet, Yindan Xinnaotong can prevent AS lesions by improving their blood lipid levels, relieving oxidative stress and inflammation [24]. Buyang Huanwu Decoction can reduce plaque area and cholesterol accumulation in ApoE^−/−^ mice, promote the differentiation of regulatory T cells by regulating the TGF-*β*/Smad2 pathway, and then play an anti-AS effect [25]. AS is more common in the middle-aged and elderly and is closely related to senescence. Deficiency in origin and excess in superficiality is the fundamental pathogenesis of AS, and the treatment of AS from the kidney has attracted the attention of many scholars. Kidney stores essence, which is the foundation of innateness. Kidney essence is the original material basis of kidney qi. Kidney qi is the qi produced by the essence of the kidney and is the basic driving force of the physiological function of the kidney. Both the yin and yang of kidney are the roots of yin and yang in the viscera. Our group made an organic connection between AS and the theory of kidney deficiency earlier in China. The results of our previous studies have shown that the compound Shoushen granules, a Traditional Chinese Medicine for tonifying the kidney, can significantly downregulate the serum lipid content of ApoE^−/−^ mice induced by a high-fat diet to regulate their lipid metabolism disorders and reduce Toll-like receptor 4 (TLR4), intercellular adhesion molecule-1 (ICAM-1), and monocyte chemotactic protein-1 (MCP-1) to improve the inflammatory response [26]. For patients with carotid atherosclerosis (CAS), the Shoushen granules can reduce the TC and TG levels in their serum, reduce their serum inflammatory factors C-reactive protein (CRP) and interleukin-6 (IL-6), upregulate the serum ApoE level, and improve their carotid intima-media thickness (CIMT), brachial-ankle pulse wave velocity (baPWV), and carotid artery elasticity [27–29]. Based on the above results, BSJZ consists of 10 g each of Tusizi, Gouqizi, Shayuanzi, Nüzhenzi, Fupenzi, and Heidou. Tusizi nourishes the kidneys and fixes essence and Gouqizi nourishes the kidneys and enhances essence, together as “Jun”; Shayuanzi nourishes the kidneys and enhances yang, Nüzhenzi replenishes the kidneys and yin, and Fupenzi nourishes the kidneys and fixes essence, together as “Chen”; Heidou promotes blood circulation and detoxifies the inner toxin as “Zuo Shi”; the combination of all the herbs has the effect of tonifying the kidney, replenishing essence, and lowering blood fat to unblock vessel. Our research further confirmed that BSJZ could significantly reduce the free cholesterol deposition in the aortic sinus of ApoE^−/−^ mice induced by a high-fat diet and regulate peroxisome proliferator-activated receptor gamma (PPAR*γ*)-LXR*α*-ABCA1 pathway to promote cholesterol efflux, thereby improving lipid metabolism disorders [9]; BSJZ can also reduce the expression of tumor necrosis factor-*α* (TNF-*α*) and inflammatory factor such as interferon-*γ* (IFN-*γ*) and interleukin-10 (IL-10) in peripheral blood serum of ApoE^−/−^ mice induced by a high-fat diet, increase the collagen fiber content of the aortic sinus in mice, enhance the level of autophagy, and effectively intervene AS [10]. However, whether BSJZ can regulate AS through circRNA requires further researches and verifications.

In this experiment, Arraystar version 2.0 mouse circRNA chip sequencing was used to obtain mouse aortic circRNAs expression profiles. Scatter plots, volcano plots, and cluster analysis plots showed significant differences in circRNAs in each group. Combine with Venny software to screen out the differentially expressed circRNA intersections between the comparison groups and construct a ceRNA network through ceRNA bioinformatics analysis to obtain aminopeptidase N (Anpep/CD13) mRNA related to circRNA. Anpep is a type II membrane metallopeptidase with 963 amino acids. As a signal transduction molecule, Anpep can be used as an enzyme to regulate peptide activity by cleaving N-terminal amino acids or to participate in biological processes such as cell phagocytosis and cholesterol uptake [30]. Recent studies have shown that Anpep is a new protein related to non-alcoholic fatty liver disease (NAFLD). In plasma samples of NAFLD patients, there was a significant correlation between Anpep protein levels and ALT levels [31]. Anpep is considered to be related to cholesterol absorption and can be regarded as a target of bexarotene to reduce NPC1L1 and CD13 mRNA expression and plasma cholesterol concentration in the aorta and intestinal epithelial cells of ApoE2-KI mice fed with a high-fat diet and reduce the cholesterol absorption in intestine, thereby improving cholesterol homeostasis and inhibiting the progression of AS [32]. Other studies have shown that Anpep is involved in proinflammatory processes, such as TLR4 signal transduction [33], receptor-mediated, dynein-dependent endocytosis of multiple receptors [34], and cell and cell-matrix adhesion [35], as well as monocyte transportation and macrophage aggregation at sites of inflammation [36]. In addition, Anpep is also a member of the renin-angiotensin system (RAS), which can regulate the expression of angiotensins 2, 3, and 4 and may play a role in the acute Stanford type A aortic dissection [37–39].

In summary, AS plaque area and lipid accumulation significantly increased in aortic sinus of ApoE^−/−^ mice induced by a high-fat diet; the results of bioinformatics analysis indicated that there were 74 differentially expressed circRNAs in group *M* (23 upregulated and 51 downregulated; *P* < 0.05, FC ＞ 1.5) compared with group C, and a total of 653 differentially expressed circRNAs in group BS (474 upregulated and 179 downregulated; *P* < 0.05, FC ＞ 1.5）compared with group M. Combined with the results of qRT-PCR, we found that mmu_circRNA_43970 was upregulated, mmu_circRNA_22187 and mmu_circRNA_32161 were downregulated in the aorta of mice in group M, compared with group C, and the further experiment exhibited that the expression of Anpep mRNA and protein in group M was higher than that in group C. After the intervention of BSJZ, the aortic sinus AS plaque area and lipid accumulation in mice were significantly reduced, and the expressions of mmu_circRNA_22187 was upregulated, Anpep mRNA and protein were downregulated, suggesting that the effective therapeutic mechanism of BSJZ on AS might be related to the regulation of Anpep by mmu_circRNA_22187.

## Figures and Tables

**Table 1:** Sequences of gene-specific primers used in RT-qPCR.

Gene	Primers
mmu_circRNA_20264	F:5′CTTTGGAGCGAGGACTCACTG3′
	R:5′GGAATGTCTGCTTGTGTTCTGC3′
mmu_circRNA_22187	F:5′TGGAGATGGATGATGAGAGCAA3′
	R:5′CGAGGGTTTCTTTCTCCTTCTG3′
mmu_circRNA_32161	F:5′AGCAGCAAAAACTCAGACAGTCA3′
	R:5′CTCCATTTCTCCTGCCATTCA3′
mmu_circRNA_43213	F:5′GCTGCTGGAAAACACAGTGC3′
	R:5′CTGGTAGACAGGCTCGCAGTAG3′
mmu_circRNA_43970	F:5′AGGATAAAATCACGATGAGCAG3′
	R:5′TTGACCACACCATTAGAGCAC3′
Anpep mRNA	F:5′CTTTCTCCTTTGCCAATCTCA3′
	R:5′CTTTGTTCTCCTTCACCCAGTC3′
GAPDH	F:5′CACTGAGCAAGAGAGGCCCTAT3′
	R:5′GCAGCGAACTTTATTGATGGTATT3′

## Data Availability

The data used to support the findings of this study are included within the article.
